# Measuring Age-Friendly Communities in New England: Promising Pilot From the New Hampshire Alliance for Healthy Aging

**DOI:** 10.1093/geroni/igaa057.167

**Published:** 2020-12-16

**Authors:** Laura Davie, Alison Rataj, Beth Dugan, Renee Pepin, Josephine Porter, Jennifer Rabalais, Matha Tecca

**Affiliations:** 1 University of New Hampshire, Durham, New Hampshire, United States; 2 University of Massachusetts Boston, Kingston, New Hampshire, United States; 3 University of Massachusetts Boston, Boston, Massachusetts, United States; 4 Dartmouth College, Lebanon, New Hampshire, United States; 5 University of New Hampshire, Concord, New Hampshire, United States

## Abstract

The New Hampshire Alliance for Healthy Aging is a statewide coalition building partnerships that support and promote healthy aging throughout the state. Through a collective impact approach, six domains (fundamental needs, living arrangements, caregiver support, social and civic engagement, physical and mental wellbeing, and advocacy) were defined to characterize and support the ongoing evaluation of age friendly communities. This poster describes a measurement framework and the development of a strategy to support gathering data across northern New England. A committee of state and national experts has convened to identify the best available indicators and measures for each of the domains and to expose gaps in available data. Representation includes individuals representing the University of New Hampshire, Tri-State Learning Collaborative on Aging (TSLCA), UMass Boston’s Department of Gerontology, and the 100 Million Healthier Lives Initiative (Institute of Healthcare Improvement). Researchers scanned national and state level sources for credibility, consistency, and availability of comparison information. Across the six domains, 43 indicators were selected. 26 did not have available data. Factors measuring social determinants of health are central and especially difficult to quantify, demanding new strategies and data collection approaches. Funding is essential for efforts to define and pilot a new data module to capture a broader set of meaningful data to measure and evaluate age friendly communities. Comprised of grassroots efforts across the fastest aging region of the country, Northern New England, under the Tri-State Learning Collaborative on Aging, is a prime location to use as a pilot project for this module.

